# COVID-19-Associated Rhino-Orbital Mucormycosis in a Tertiary Health Care Center in Odisha, India

**DOI:** 10.7759/cureus.43811

**Published:** 2023-08-20

**Authors:** Souvagini Acharya, Sulin K Behera, Somy Purohit, Alaka Sahu, Braja B Panda, Sharmistha Behera

**Affiliations:** 1 Department of Otolaryngology, Veer Surendra Sai Institute of Medical Sciences and Research, Sambalpur, IND; 2 Department of Microbiology, Veer Surendra Sai Institute of Medical Sciences and Research, Sambalpur, IND; 3 Department of Ophthalmology, Veer Surendra Sai Institute of Medical Sciences and Research, Sambalpur, IND; 4 Department of Pathology, Veer Surendra Sai Institute of Medical Sciences and Research, Sambalpur, IND; 5 Department of Radiodiagnositic, Veer Surendra Sai Institute of Medical Sciences and Research, Sambalpur, IND

**Keywords:** retrobulbar amphotericin b, liposomal amphotericin b, diabetes mellitus, mucormycosis, covid-19

## Abstract

Background

Mucormycosis is an opportunistic infection that mainly affects immunocompromised individuals, including those with uncontrolled diabetes, malignancies, or those who have exposure to high-dose corticosteroids for a long time. Western Odisha, India, witnessed a significant rise in post-COVID-19 rhino-orbital mucormycosis (ROM), which created a need for comprehensive research on post-COVID-19 ROM.

Objective

This study aimed to investigate the prevalence, clinical characteristics, and outcomes of post-COVID-19 ROM in a tertiary care hospital in Western Odisha, India, with the objective of understanding ROM as a nationally notifiable disease.

Subjects and methods

A prospective hospital-based study was conducted. Mucormycosis cases were reported within the period, from May 17, 2021, to July 31, 2021, including all post-COVID-19 patients who exhibited clinical manifestations of mucormycosis. Patients with histopathologically negative reports of mucormycosis were excluded.

Results

Of the 35 included, 25 were diagnosed with ROM. The age group of 46-50 years showed a predominance (n=6), with a mean age of 50.53 years. The male-to-female ratio was 2:1. Specifically, 88% of the patients included had diabetes mellitus, 8% had chronic kidney diseases, 8% had sepsis, and 4% had hypertension. ROM was the predominant manifestation (60%, n=15), with the majority presenting with orbital cellulitis (80%), followed by unilateral orbital apex syndrome (12%), bilateral orbital apex syndrome (4%), ophthalmic vein involvement (4%), and osteomyelitis of the maxillary sinus (4%). Irrigation of the wound was performed, and all 25 ROM patients received IV liposomal amphotericin B (L-AMB).

Conclusion

Post-COVID-19 status with elevated blood sugar levels was a major risk factor for ROM. Early diagnosis, debridement, L-AMB, retrobulbar AMB deoxycholate, and exenteration are the possible solutions.

## Introduction

The COVID-19 virus SARS-CoV-2 emerged in 2019, and its spread was classified as a global pandemic by the World Health Organization (WHO) in March 2020 [1]. Since then, the world has experienced multiple waves of the COVID-19 pandemic, including in developing countries like India. The clinical manifestations in all of these waves have remained more or less consistent, with changes in infection intensity. Of all the waves, the second wave was considered the most severe [2]. Because of the accelerating development of symptoms in the second wave, the use of systemic corticosteroids was widespread to decrease inflammation [3]. The excessive use of steroids resulted in opportunistic infections, the most notable of which were rhino-orbital mucormycosis (ROM) and rhino-cerebral mucormycosis. COVID-19 patients are given substantial doses of steroids to reduce the inflammatory response, weakening their immune system and making them more vulnerable to mucormycosis [4]. Steroids are known to increase blood sugar levels through various mechanisms, creating an acidic environment that promotes fungal growth. Steroids aid hepatic gluconeogenesis, reduced the absorption of glucose, and also decreased uptake by the various cells of the body. It is estimated that in India, mucormycosis had a pre-COVID-19 incidence of 0.14 per 1,000, but it became a pandemic after COVID-19 [5].

Mucormycosis is an opportunistic infection that affects immunocompromised people, particularly the diabetic population. The fungus is common and can be found in the soil and air. These spores can form hyphae in the paranasal sinuses and spread to the orbit and brain via anatomical connections [6]. Several studies have found that patients with diabetes mellitus are more likely to develop COVID-19 and, subsequently, mucormycosis. COVID-19 morbidity in diabetic patients may be caused by a loss in T-cell activity, a decrease in viral clearance, immunological suppression, or a high cytokine storm [7-10]. Therefore, diabetic patients are at an increased risk of developing mucormycosis.

Mucormycosis in various organs can result in the following manifestations but are not limited to sinusitis, numbness in the face, teeth-loosening problems, jaw problems, vision loss, blurred vision, diplopia, skin necrotic lesions, thrombosis, lung-worsened respiratory symptoms, or chest pain [11]. Inside the human body, mucormycosis infection begins in the nasal or maxillary sinus and progresses to the ethmoid or sphenoid sinus. Then, it reaches the orbit via the nasolacrimal duct, the ethmoid foramina, or by splitting the lamina papyracea [12]. When the hyphae invade the blood vessels, it causes dry gangrene in the eye. When angioinvasion occurs in the internal carotid artery or cavernous sinus, cerebral infarction occurs. It can also cause aneurysms, mycotic abscesses, and hematogenous dissemination [13]. Angioinvasion can also result in ocular artery necrosis, which can lead to blindness, cranial nerve palsies, or sensory-motor impairments [11].

In those with diabetes mellitus, the CNS is the third most typical spread site. When an infection spreads directly from the paranasal sinuses or indirectly through hematogenous dissemination, cranial infection results [14]. The fungal hyphae develop in the internal elastic lamina of the blood vessel and invade the arterial lumen to cause intravascular thrombosis [15]. This causes vascular occlusion and results in hemorrhagic necrosis and brain tissue infarction even before the fungus invasion into neuronal tissues. When the fungus causing advanced CNS mucormycosis invades the necrotic brain parenchyma, death may result [16,17].

This study aimed to understand post-COVID-19 mucormycosis to alert the public about the necessity for treatment on a national and international scale.

## Materials and methods

The study complied with all the ethical standards stated in the 1964 Declaration of Helsinki. The approval for the conduct of the study was obtained from the institutional ethical review board. The study was approved by the institutional ethics review board (Institutional Ethics Committee; Letter Number: 141-2022/I-F-O/03/ Dt.05.08.2022). All of the patients who opted to take part in the research study gave their informed consent.

This hospital-based prospective study was conducted from May 17, 2021, to July 31, 2021, at a Tertiary Care Hospital in Western Odisha. Since the first case was hospitalized on May 17, 2021, a total of 35 patients were seen. The month of May saw a rise in COVID-19 cases along with a new epidemic called mucormycosis infection. Every effort was made to form a multidisciplinary committee. It included medical professionals from a variety of specializations, including pathology, pharmacology, dermatology, pulmonary medicine, neurosurgery, dental surgery, ear-nose-throat (ENT), and ophthalmology.

Out of the 35 patients, ROM was present in 25 cases. A total of 35 patients were selected, those who satisfied the inclusion criteria. Patients with COVID-simulating symptoms and/or post-COVID-19 status were included. Patients older than 18 years of age were included. Those with histopathologically negative results for mucormycosis were excluded from the study. All patients underwent thorough medical, otorhinolaryngeal, and ophthalmological evaluation along with history taking. The steps involved in the study were history taking, clinical diagnosis, microbiological study with potassium hydroxide (KOH) mount, and Sabaroud dextrose agar (SDA) culture, histopathological study, high-resolution CT scan (HRCT), magnetic resonance imaging (MRI), brightness scan (B-scan), electrocardiography (ECG), and echocardiography (ECHO). More than 50% of patients underwent diagnostic nasal endoscopy (DNE) except one who was too sick to undergo surgical treatment. The patients' specimens (nasal swabs or orbital tissues) were collected in two separate clean tubes, one with and one without 10% formalin, and then processed. The samples were tested using direct KOH analysis and fungal culture. The specimens were inoculated in a pair of tubes containing SDA and incubated at 37°C and 25°C, respectively. The cultures were inspected for growth on a daily basis. Conventional procedures such as the lactophenol cotton blue (LCB) mounts were used to determine the fungal isolates. Sections of tissue were stained with eosin and hematoxylin, periodic acid-Schiff, and Grocott methenamine silver stain for histological evaluation. The treatment employed in the study included a multi-disciplinary team approach with the administration of amphotericin B (AMB) deoxycholate, surgical debridement, and exenteration. The surgical management employed in the patients was primarily modified Denker’s method [18].

Transcutaneous retrobulbar amphotericin B

Fifty mg of amphotericin B deoxycholate was reconstituted with 14 ml of distilled water [17]. This resulted in 3.5 mg of amphotericin B deoxycholate in 1 ml. It was then administered transcutaneously in the inner third of the lower orbital rim with the help of a 24-gauge needle directed toward the medial orbital wall. The amphotericin B deoxycholate solution was injected consecutively for five days or on alternate days, particularly in cases showing inflammation post-injection. The reconstituted solution can be kept under refrigeration at three to five degrees Celsius and can be used for a week. This solution can also be used for the local irrigation of wounds.

## Results

With the advent of post-COVID-19-associated mucormycosis, a similar trend was seen in the western part of Odisha. A total of 35 patients were identified with mucormycosis and were included in the study. Of the 35 admitted patients, 25 had characteristics of both rhino- and orbital mucormycosis. The remaining 10 patients had other forms of mucormycosis that are pulmonary, rhino-cerebral, and cutaneous mucormycosis. All patients belonging to the age group 46 to 50 years had a greater association with ROM (n = 6), and the mean age was found to be 50.53 years. The male-to-female ratio was 2:1, with 68% of the patients being male. 

The signs and symptoms reported by the patients were consistent with the area infected. A list of the common signs and symptoms reported has been presented in Table 1. The patients were then subjected to radiological investigations such as MRI/MRI angiography (brain, paranasal sinuses, orbits) and CT scan. Of the patients who underwent MRI scans, two patients had cavernosal involvement, and three patients had a frontal abscess. Of all the ROM cases, 80% were orbital cellulitis, 12% were unilateral apex syndrome, 4% were bilateral apex syndrome, 4% had ophthalmic vein involvement, and 4% had osteomyelitis of the maxillary sinus.

**Table 1:** Common signs and symptoms reported by the patients

Symptoms	Number of patients
Eye swelling	16
Facial pain	11
Decreased vision	10
Headache	07
Black discoloration of the skin of the nose	03
Painful eye movement	02
Protrusion of the eyeball	02
Disorientation to time and place	02
Toothache	02
Stuffy nose	01
Decreased sensation of alveolus	01
Pain behind the eye	01
Signs	
Orbital cellulitis	20
Periorbital edema	09
Nasal discharge	06
Tenderness in the face or eye	05
Proptosis	04
Restriction of eye movement	03
Skin eschar	03
Dental eschar	02
Ptosis	02
Nasal eschar	02
Conjunctival chemosis	02
Palatal perforation	02
Fungal elements in sinuses	02
Shock	02
Erythema of face	01

Upon assessment of the patient details, the most common comorbidity among the patients was diabetes mellitus (88%), followed by chronic kidney disease (8%), sepsis (8%), and hypertension (4%). Forty-four percent of the patients reported having a previous history of hospitalization, 32% were on oxygen therapy, and 28% had a history of steroid administration.

**Figure 1:**
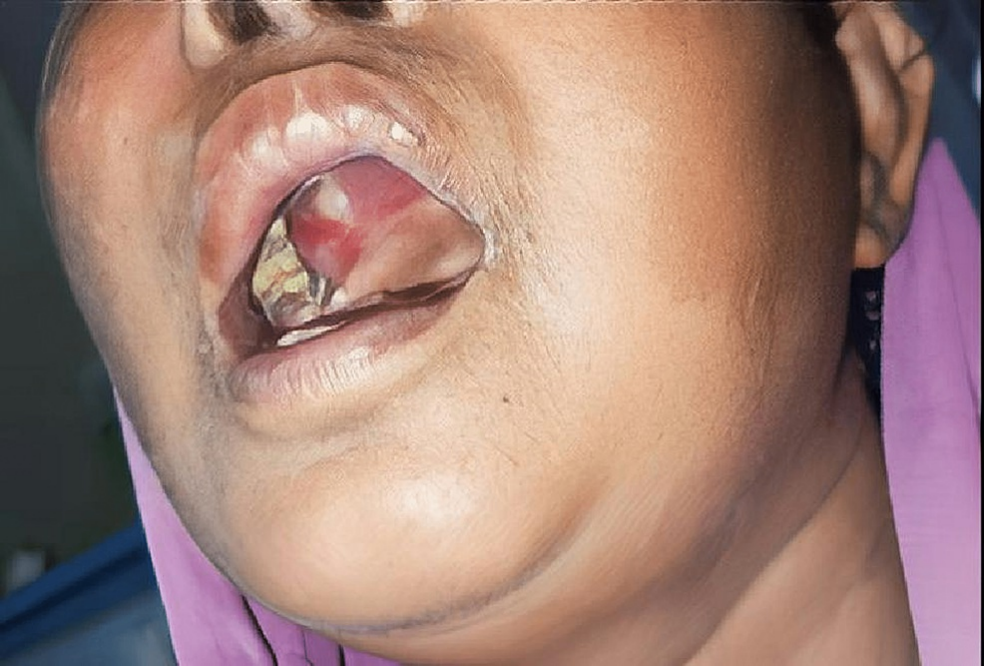
Palatal involvement

**Figure 2:**
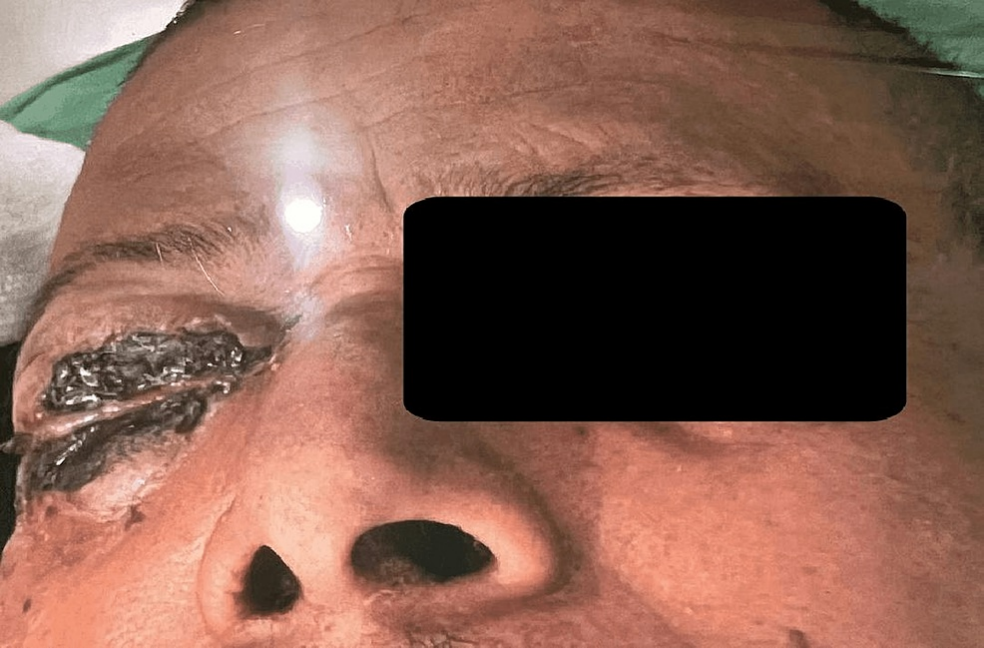
Post-COVID-19 mucormycosis patient with orbital involvement and eschar over both lids

**Figure 3:**
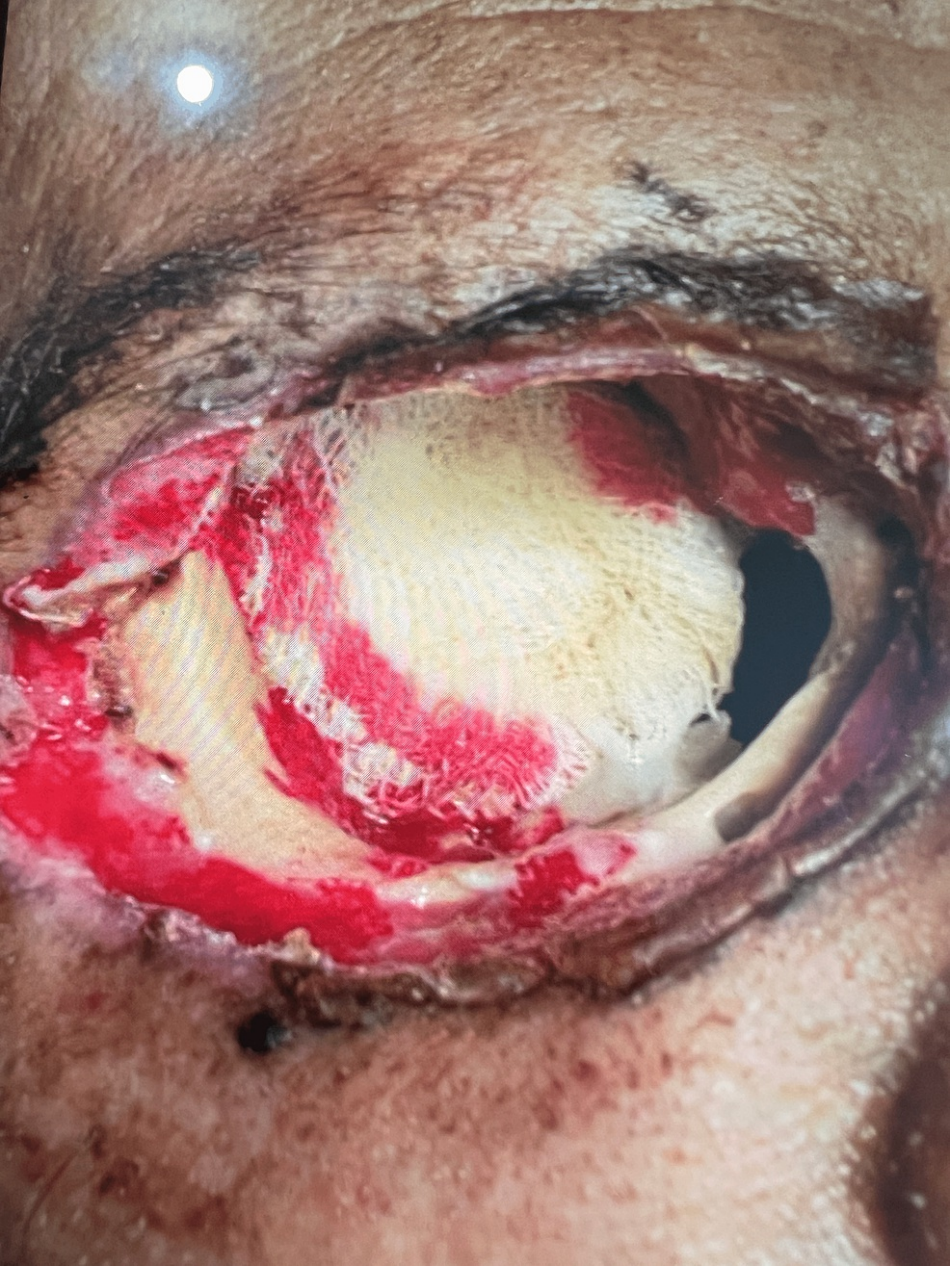
Right orbit exenteration

**Figure 4:**
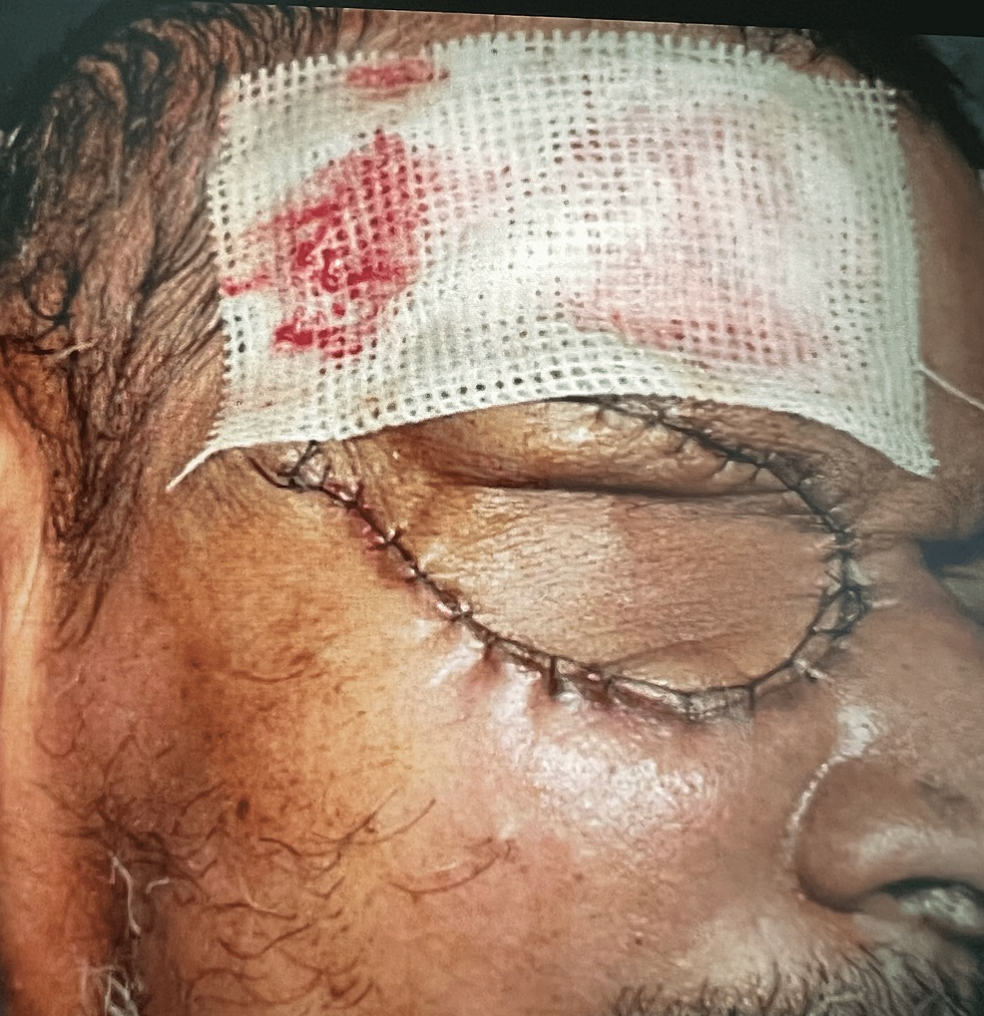
Orbital reconstruction with rotational flap done

Most of the patients (>50%) underwent DNE, except one who was too sick to undergo surgical treatment. Furthermore, microbiology and histopathology samples were sent post-DNE. The KOH mount of 15 patients was suggestive of mucormycosis and showed the growth of fungal hyphae. Also, eight patients showed fungal growth in the SDA medium. Additionally, in the histopathologic examination, seven patients showed changes suggestive of mucormycosis. Sixty-eight percent of the patients (n = 17) underwent surgical procedures within one to two days of admission, provided that they were fit for surgery. Of the 17 patients, ten underwent functional endoscopic sinus surgery, three underwent abscess drainage, one underwent middle meatal antrostomy with middle ethmoidectomy, one underwent septoplasty with surgical debridement, two underwent a modified Denker's procedure, and, finally, two underwent exenteration of the right eye with rotational flap replacement. The patients who underwent surgical debridement were then followed up with alternate-day suction and also amphotericin B nasal irrigation (Figures 1-4).

 

All patients (n = 25) were administered IV liposomal amphotericin B, and 24 patients received transcutaneous retro-orbital amphotericin B deoxycholate, and amphotericin B irrigation of the wounds was done for all 25 patients. During the conduct of the study, four patients were lost to follow-up/death, two patients were referred to a higher center, and 19 patients were discharged on request after feeling better.

## Discussion

We conducted a single-center prospective study of 35 patients with COVID-19-associated mucormycosis. The pre-COVID-19 prevalence of mucormycosis was 0.14 cases per 1,000 patients and rose to seven cases per 1,000 patients. The latter value was found to be 50 times higher than the former recorded value [19]. As per the findings of our study, there was a greater incidence of ROM among males and in the age group 46-50 years (24%). A similar pattern was established in the study conducted by Ramaswami et al. [20] where the median age of patients diagnosed with COVID-19-associated mucormycosis was 44.5 years and was more common (60%) among the males. Roden et al. [21] have hypothesized that the presence of circulating estrogen within the female system can have a protective action over systemic fungal invasions, thus the lower incidence in females.

According to experts, it is believed that comorbid diseases such as diabetes and the excessive use of steroids could have contributed to the epidemic of mucormycosis among COVID-19 patients [22]. In comparison with the prevalence of mucormycosis in the comorbid population, the results of our study demonstrate that 88% of the patients were diabetic. This aligns with the findings from studies conducted by Corzo-León et al. [23] and Dolatabadi et al. [24]. As per their findings, diabetes mellitus was a notable predisposing factor in mucormycosis patients, with an overall prevalence of 72% and 75.4% in the Mexican and Iranian populations, respectively. However, the role of diabetes mellitus as a significant risk factor is less expressed in the European population: 18% in Italy, 23% in France, and 29% in Greece. This suggests that there are other risk factors such as cancer and particularly hematological malignancies in the development of mucormycosis [25,26]. In India, there has been widespread indiscriminate use of steroids to control the inflammatory onset caused due to COVID-19 infection, even in mild cases. The underlying reasons include non-evidence-based practice, inadequate monitoring, availability of over-the-counter steroids, and lack of public awareness [27]. Other comorbid conditions reported in our study are hypertension, chronic kidney disease, and sepsis.

The average date of COVID-19 infection and the reporting of mucormycosis in our study was found to be approximately 34 days. Nevertheless, a faster rate of diagnosis (14.59 ± 6.88 days) after the COVID-19 infection was observed in the meta-analysis conducted by Hussain et al. [19]. In another study conducted by Ramaswami et al. [20], a median age of 20 days was reported between the onset of COVID-19 symptoms and the onset of COVID-19-associated mucormycosis symptoms. Additionally, in our study, only 44% of the patients were previously hospitalized and 32% had oxygen support.

The patterns of mucormycosis may vary among patients with different risk factors. Palatal necrosis is a hallmark feature in patients with mucormycosis [28]. However, only two patients were reported to have a palatal perforation in our study. Other specific signs to look for while analyzing for mucormycosis include cranial nerve involvement, diplopia, periorbital edema, proptosis, palatine ulcer, and sinus pain [23]. In this study, nine patients reported periorbital swelling, and four of them had proptosis. Also, a portion of the patient reported cutaneous involvement in the presentation of mucormycosis such as black discoloration of the skin and skin eschar. Eighty percent of all ROM cases involved orbital cellulitis, 12% involved unilateral or bilateral apex syndrome, 4% involved ophthalmic vein involvement, and 4% involved maxillary sinus osteomyelitis. According to recommendations, contrast-enhanced CT scans were used to determine the extent of the infection after the microbiological diagnosis was verified by KOH-calcofluor mount showing aseptate hyphae [29]. In our study, 80% of the patients reported orbital cellulitis, and a similar trend was seen by Cornely et al. [29], with around 60% of the mucormycosis patients reporting orbital cellulitis. Moreover, in the study conducted by Ravani et al. [30], 62.2% of the patients underwent surgery, and our study also demonstrates a pattern of 68%.

All 25 patients had intravenous liposomal amphotericin B, and 24 patients with orbital involvement and/or visual loss received retro-orbital amphotericin B deoxycholate, followed by one month of oral posaconazole. We lost four of the critically ill patients who were admitted to us under the proper procedure, and two others were sent to higher centers. Along with the other patients who passed away from comorbidities, one had a large eschar and was admitted with post-COVID-19 mucormycosis. The remaining patients are on the road to recovery, although naturally they still have comorbid conditions from the fungus invasion. Amphotericin B can be nephrotoxic; hence, it is important to constantly monitor patients' electrolytes and renal function tests [20].

This study had a few limitations. The authors were unable to identify the type of corticosteroids used (methylprednisolone, dexamethasone, prednisolone, hydrocortisone, budesonide, etc). Furthermore, the route of steroid administration (inhalational or systemic) and the duration of steroid use could have been analyzed. The complications related to steroid use such as hyperglycemia and subsequent insulin use could have shed more light on the onset of mucormycosis in the diabetic population. Another limitation of our study is that it was conducted at a single center and had a very small sample size. Since the sample size was too small which included both patients having concurrent COVID-19 symptoms and those having post-COVID-19 ROM, we could not differentiate between the characteristics within the two groups, which is only possible in case of a larger sample size. 

## Conclusions

All patients referred to or presenting to the ophthalmologist with vision problems or eye complaints with or without a history of concomitant uncontrolled diabetes mellitus must have a high index of suspicion of ROM in the COVID-19 period. The figures might only be at the very tip of the iceberg. The risk factors and document management modalities require more research. Diabetes mellitus and the use of corticosteroids in the treatment of COVID-19 are two major risk factors that should be thoroughly investigated. Therefore, it is crucial to maintain ideal glucose levels and utilize corticosteroids sparingly to lessen the severity of mucormycosis in post-COVID-19 patients. Reduced disease progression and a lower mortality rate can be achieved with early diagnosis and therapy.
